# Neurotrophin signalling in the human nervous system

**DOI:** 10.3389/fnmol.2023.1225373

**Published:** 2023-07-04

**Authors:** Sarah Ateaque, Spyros Merkouris, Yves-Alain Barde

**Affiliations:** School of Biosciences, Cardiff University, Cardiff, United Kingdom

**Keywords:** Neurotrophins (NTs), Trk receptors, memory, depression, neurodegeneration, blood platelets, BDNF ELISA

## Abstract

This review focuses on neurotrophins and their tyrosine kinase receptors, with an emphasis on their relevance to the function and dysfunction in the human nervous system. It also deals with measurements of BDNF levels and highlights recent findings from our laboratory on TrkB and TrkC signalling in human neurons. These include ligand selectivity and Trk activation by neurotrophins and non-neurotrophin ligands. The ligand-induced down-regulation and re-activation of Trk receptors is also discussed.

## Introduction

The discovery of nerve growth factor (NGF), the mother of all growth factors, was a historical achievement. As early as 1960, a secretory protein was shown to be essential for the survival of neurons during the development of the nervous system (Cohen, 1960; Levi-Montalcini and Booker, 1960; Levi-Montalcini, 1964). Remarkably, NGF was later shown to be synthesized in limiting amounts by tissues innervated by NGF-dependent neurons thereby allowing target tissues to dictate the density of their own innervation (Korsching and Thoenen, 1983; Edwards et al., 1989). Both the survival promoting activity and the retrograde mode of action were subsequently found to also apply to the 3 other members of the NGF family, brain-derived neurotrophic factor (BDNF), Neurotrophin-3 (NT3) and Neurotrophin-4 (NT4, reviewed in Huang and Reichardt, 2001). As it turned out, these properties are limited to neurons of the peripheral nervous system (PNS) and the degree to which they may also apply to the central nervous system (CNS) is unclear. BDNF is the most widely expressed neurotrophin in the CNS, but it is not a major survival factor for CNS neurons in the absence of lesions such as axotomy (Sendtner et al., 1992; Yan et al., 1992; Di Polo et al., 1998). A mouse brain developing in the absence of BDNF is measurably smaller and lighter than a wild-type control, but it nonetheless closely resembles a wild-type brain, with no major neuronal populations missing (Rauskolb et al., 2010). However, the behavior of such mutant animals is profoundly affected and they fail to move much at all (Rauskolb et al., 2010). As endogenous BDNF is transported anterogradely and stored in pre-synaptic secretory vesicles (Dieni et al., 2012), its mode of action primarily fits a feedforward model, with BDNF modulating the properties of postsynaptic neurons. Rather than an essential target-derived survival factor, BDNF thus acts as an anterograde messenger in the CNS, displaying many of the properties of classical neurotransmitters.

The following sections deal mostly with BDNF, its role in memory-related processes and in mood disorders. As the minuscule quantities of endogenous BDNF in the brain have hampered the delineation of its physiopathology as well as a clear understanding of its cell biology, quantitative aspects are also discussed, including post-mortem measurements of BDNF levels in Alzheimer’s disease. Last, the review deals with the BDNF receptor TrkB and its relative promiscuity, its activation by non-neurotrophin ligands and the prevention of its down-regulation typically observed following exposure to saturating ligand concentrations. The role of neurotrophins and their receptors in cancer is not discussed (for a recent review, see Pfaff et al., 2021).

### BDNF and TrkB: quantitative considerations and distribution

In the brain, BDNF levels increase significantly after birth in mammals as a result of developing connectivity and increased neuronal activity, the major driver of BDNF expression in neurons (Zafra et al., 1990; Patterson et al., 1992; Hong et al., 2008). In the developing visual system, unilateral section of the optic stalk before innervation of the tectum, or injection of tetrodotoxin into one eye, markedly delays the increase of *Bdnf* expression in the tectum compared with the contralateral side (Herzog et al., 1994). Given the very low abundance of BDNF even in the adult brain, a reliable detection of BDNF by Western blot remained difficult for a long period of time. It is only recently that a specific monoclonal antibody suitable for Western blot detection became available and its specificity validated using brain extracts prepared from mice lacking *Bdnf* (Andreska et al., 2020; Wosnitzka et al., 2020). Likewise, the cellular and sub-cellular distribution of endogenous BDNF and of its mRNA remain challenging, both on brain sections and in cultured neurons (Dieni et al., 2012; Will et al., 2013). The straightforward notion that the *Bdnf* mRNA is primarily localized in neuronal cell bodies, as opposed to axons and dendrites (Will et al., 2013), and that the BDNF protein primarily accumulates in presynaptic terminals (Dieni et al., 2012) is not universally shared and has been challenged by a number of studies relying on overexpression paradigms. Some of these studies include GFP-tagged versions of BDNF, under the assumption that the tag would report the distribution of the endogenous protein (Egan et al., 2003; Matsuda et al., 2009; Leschik et al., 2019). However, large tags of this kind interfere with the processing and secretion of BDNF in transfected cells (Wosnitzka et al., 2020), making it unlikely that such tools reflect the distribution of the endogenous protein. Contrasting with the very low levels of BDNF in the brain, its receptor, the tyrosine kinase TrkB, can be readily detected. TrkB is not only expressed by most neurons in the CNS but also at levels far higher than those of BDNF. RNAseq data indicate that in the adult human brain, the ratio of *NTRK2*, the gene encoding TrkB, to *BDNF* is about 25–30 to 1 in sub-regions of the hippocampus, the visual and prefrontal cortex and more than 500 to 1 in the parietal cortex. The second endogenous TrkB ligand NT4 does not account for the ligand-receptor imbalance as NT4 is barely detectable in the adult CNS.^1^ Observations indicating that TrkB can be activated by non-neurotrophin ligands, including antidepressants (see below), may contribute to explain the quantitative discrepancies between the levels of TrkB and those of BDNF. In addition, zinc, various G-protein coupled receptor agonists as well as EGF receptor ligands have all been shown to activate TrkB (Lee and Chao, 2001; Huang et al., 2008; Puehringer et al., 2013; Zagrebelsky et al., 2018; Casarotto et al., 2021).

### BDNF: the Val/Met polymorphism

The notion of neurotrophin limited availability, however technically inconvenient and challenging, is a key aspect of BDNF’s physiology, just as is the case with NGF in the PNS (see Introduction). This notion was supported early on by observations made with brain slices prepared from mouse mutants. The loss of just one *Bdnf* allele was found to cause an impairment of long-term potentiation (LTP) of a magnitude comparable to the loss of both *Bdnf* alleles (Korte et al., 1995). This important result was later extended to humans: a chromosomal inversion silencing one *BDNF* allele causes cognitive dysfunction and abnormal weight gain in the affected individual (Gray et al., 2006). Even if BDNF turned out not to be a major survival factor for developing CNS neurons (see above), it profoundly affects the extension of dendrites as well as the number of spines on dendrites (McAllister et al., 1995; Tyler and Pozzo-Miller, 2003; Rauskolb et al., 2010; Zagrebelsky et al., 2018). Given the notion that BDNF is implicated in synaptic plasticity in rodents (for a recent review, see Costa et al., 2022 and references therein), it was of particular interest to explore the relevance of these findings to the function of the human brain. In this regard, the discovery of a polymorphism in the human *BDNF* gene correlating with mild memory impairments was a key development (Egan et al., 2003). This missense mutation, designated rs6265, causes a replacement of valine (Val) by methionine (Met) in position 66 in the BDNF precursor pro-BDNF (Egan et al., 2003). The association of this polymorphism with modest impairments of episodic memory was later independently confirmed (Cathomas et al., 2010). While the biochemical impact of the Val → Met is still not entirely clear, it has been noted that the Met pro-peptide binds with higher affinity to the mature protein than the Val pro-peptide (Uegaki et al., 2017). Also, *in vitro* results obtained with a mouse model engineered to replicate the human BDNF polymorphism indicate a reduced secretion of BDNF from cultured neurons (Chen et al., 2006). In addition, the steady state levels of BDNF in adult mutant animals are decreased in the hippocampus (Bath et al., 2012), but not in whole brain lysates (Chen et al., 2006) whereby the reasons for this differential effect are not clear. While Val → Met mutant mice do not show clear memory deficits, they display an anxiety-type phenotype that cannot be ameliorated by the administration of the antidepressant fluoxetine (Chen et al., 2006; Bath et al., 2012). This finding is of special interest given recent observations that antidepressants potentiate TrkB activation by BDNF (see below). Regarding the incidence of the BDNF polymorphism in humans, it is of note that the frequency of the Met66 allele varies considerably across populations, from 0 to 72% (Petryshen et al., 2010). The polymorphism is virtually absent in all sub-Saharan African and some American indigenous populations, but dominant in various Asian ethnic groups (Petryshen et al., 2010). In addition, it has been noted that there are 3 additional protein-encoding genes, designated *LIN7C*, *LGR4*, and *CCDC34,* in the 500 kB region on Chromosome 11 that include the *BDNF* gene (Petryshen et al., 2010), itself spanning around 70 kb (Cattaneo et al., 2016). Both the close proximity of these genes and the geographical distribution of the rs6265 polymorphism caution a direct causality between the *BDNF* polymorphism and behavioral alterations in humans. These observations also emphasize the importance of animal models carrying a mutation limited to the *Bdnf* gene (Chen et al., 2006). Meanwhile, a second mouse Val → Met mutant has been generated (Warnault et al., 2016) as well as a rat mutant, the latter showing a selective impairment of fear memory (Jaehne et al., 2022). BDNF measurements in hippocampal lysates of these rats indicate no differences between Val/Val and Met/Met homozygote animals, neither in males nor in females (Jaehne et al., 2022).

In the context of phenotypes associated with the Val → Met polymorphism, it is also worth noting that Met/Met mice gain weight abnormally when placed on a high fat diet (Xie et al., 2023). These findings fit well with the notion that the release of BDNF may be decreased in these animals (see above) and extensive mouse work has mapped in some detail the hypothalamic circuitry involved in body weight regulation by BDNF/TrkB signalling (for review, see Xu and Xie, 2016). A slight reduction of TrkB activation by BDNF compromises the anti-orexic function of BDNF as noted in a recent mouse model in which a 22-amino acid carboxy terminal tag has been added to Val/Val alleles of BDNF (Wosnitzka et al., 2020). This genetic manipulation leads to a modest impairment of TrkB activation and results in a progressive weight gain that becomes measurable by about 6 months (Wosnitzka et al., 2020). In humans, the impact of BDNF and TrkB mutations on body weight regulation have been well documented (Yeo et al., 2004; Sonoyama et al., 2020), thus confirming that the availability of BDNF is a major component of body weight regulation.

In sum, this body of work indicates that BDNF levels in the CNS are critical and that even a modest decrease of BDNF/TrkB signalling in humans impacts memory performance and body weight regulation.

### BDNF/TrkB signaling in human brain dysfunction and neurodegeneration

Extensive work with rodent models has long suggested that decreased BDNF/TrkB signaling may cause CNS dysfunction in humans and BDNF is often regarded as an endogenous neuroprotectant (recently reviewed in Azman and Zakaria, 2022). In addition to the *BDNF* haploinsufficiency mentioned in the above, exome-sequencing of 197 patients with developmental and epileptic encephalopathy of unknown origin revealed that 5 of these patients have missense mutations in *NTRK2*, including a Tyr434Cys mutation in 4 of them (Hamdan et al., 2014). The results of this study also indicate dominant inheritance. With regard to age-related forms of neurodegeneration and Alzheimer’s disease in particular, it has been noted that the progression of cognitive decline monitored over a period of 6 years negatively correlates with the levels of *BDNF* mRNA quantified in the dorsolateral prefrontal cortex, in a large postmortem analysis involving over 500 patients (Buchman et al., 2016). However, BDNF/TrkB variants have not been identified thus far as risk factors associated with Alzheimer’s disease in human genetic studies (de Rojas et al., 2021). Beyond Alzheimer’s disease, impairment of BDNF/TrkB signaling is also likely to play a role and other conditions. Largely based on work in rodent models, it would seem likely that a compromised anterograde transport of BDNF from the cerebral cortex to the basal ganglia may play a role in the pathogenesis of Huntington’s disease (for review, Saudou and Humbert, 2016). Likewise, the failure of TrkB intracellular transport caused by decreased levels of dopamine may play an aggravating role in Parkinson’s disease (Andreska et al., 2023). In line with this, post-mortem examination of the brain of Parkinson’s disease patients revealed abnormal intracellular clusters of TrkB (Andreska et al., 2023).

### Depression and BDNF as a potential biomarker

With growing evidence in rodent models for a role of BDNF in synaptic plasticity in the adult CNS, attention progressively focused on the possible role of BDNF/TrkB signalling in mood disorders and especially depression (Duman, 2002). Classical antidepressants take a long time to act, suggesting that processes other than the blockade of neurotransmitter re-uptake may be at work such as synaptic remodeling (Castren and Monteggia, 2021). Early findings also associated chronic stress with decreased levels of both BDNF and TrkB while by contrast, electroconvulsive therapies and long-term administration of antidepressants correlate with increased TrkB and BDNF levels (Nibuya et al., 1995). Additional key findings comprised the discovery of a link between BDNF and the serotoninergic system (Mamounas et al., 1995) and the observation that BDNF injections into rat brains improve depressive-like symptoms (Siuciak et al., 1997). Importantly, the activation of TrkB was shown to be required for antidepressants to work (Saarelainen et al., 2003). With regard to human genetics, the expected association of the Val/Met polymorphism with the frequency or intensity of depressive episodes has been intensely scrutinised, but failed to convincingly materialize (Castren and Monteggia, 2021). By contrast, several human genetic TrkB polymorphisms were found in patients presenting a lifetime history of depression and suicide attempts (Kohli et al., 2010). The role of TrkB in depression has received additional support following the astonishing observation that all anti-depressants bind to TrkB, in spite of a striking lack of structural relatedness among some of them (Casarotto et al., 2021 and see below). This work has been further extended with the observation that psychedelics bind to TrkB, with an affinity even higher than conventional antidepressants (Moliner et al., 2023).

Given the presence of readily measurable levels of BDNF in human serum, a very large number of studies have attempted to correlate the levels of BDNF with depression. The overall conclusion of metanalyses is that a weak inverse correlation seems to exist between the severity of depressive episodes and the levels of BDNF in serum (Tiwari et al., 2022). Yet it remains difficult to understand the logic of the assumption that BDNF levels in serum may reflect those in the brain, given that it has long been recognized that the main source of BDNF in human serum are circulating blood platelets (Yamamoto and Gurney, 1990). Furthermore, the levels of BDNF in human platelets are far higher than those in the brain, a consequence of the expression of the *BDNF* gene at comparatively high levels by mature human megakaryocytes (Chacon-Fernandez et al., 2016). The generation of serum involves blood coagulation, a process during which platelets release their content, including BDNF. Platelets are short-lived and their protein content decrease with their age (Allan et al., 2021). As physical exercise can change the composition of blood platelets and include a higher proportion of larger, younger platelets (Heber and Volf, 2015), it is conceivable that the weak correlation observed between depressive states and serum levels of BDNF may merely reflect different degree of physical activity between patient and control groups. The unquestioned benefits of physical exercise on mood and mental health may nonetheless involve BDNF, but rather as a result of the modulation of its levels in the brain by mechanisms involving metabolic intermediates such as beta-hydroxybutyrate (Sleiman et al., 2016) or the muscle-derived cytokine irisin (for review, see Jo and Song, 2021). Consistent with the lack of expression of the *Bdnf* gene in mouse megakaryocytes (Chacon-Fernandez et al., 2016), BDNF levels in mouse serum are very low, about 3 orders of magnitude lower than in human serum whereby validated BDNF measurements in mouse serum have only been made recently possible following the development of a highly sensitive BDNF ELISA (Want et al., 2023a)^2^. Furthermore, in mouse blood, BDNF levels were found to be identical in plasma compared with serum, unlike is the case in primates (Want et al., 2023a). A main source of BDNF in mouse blood is the skeletal musculature (Fulgenzi et al., 2020) and this muscle-derived BDNF has been shown to trigger the release of insulin from pancreatic β cells expressing a truncated form of TrkB (Fulgenzi et al., 2020 for a recent review on trunacted forms of TrkB, see Tessarollo and Yanpallewar, 2022). Whether or not serum levels of BDNF correlate with mouse models of depression, or indeed with any mouse model of CNS dysfunction, can now be investigated, given that the confounding factor resulting from the presence of platelet-derived BDNF is absent in the mouse. The availability of a highly sensitive BDNF ELISA also opens the possibility to perform measurements of BDNF levels in more relevant body fluids than has been the case so far, including in particular the cerebrospinal fluid and the aqueous humor. With regard to possible functions of BDNF in human platelets, recent work suggests a neuroprotective role after nerve lesion. Using a mouse line engineered to express BDNF in megakaryocytes and consequently in platelets (Dingsdale et al., 2021), the retraction of dendrites of retinal ganglion cells caused by lesions of the optic nerve has been observed to be retarded in mice containing BDNF in platelets compared with wild-type animals (Want et al., 2023b). These results suggest that in humans, the impact of lesions of the nervous system may be attenuated by the release of BDNF from platelets accompanying blood coagulation, a contribution that would be missed in traditional mouse models of nerve lesion. Together with the notion that physical exercise changes the composition of blood platelets (see above), it is thus conceivable that in humans, the benefits of exercise in conditions such as glaucoma (Tribble et al., 2021) may be explained by an increased availability of BDNF originating from platelets.

### Trk signalling in cultured human neurons

#### Expression of Trk receptors by human neurons derived from embryonic stem cells

While the *in vitro* generation of neurons from human embryonic stem (ES) cells has long become a standard procedure, questions remain as to whether populations of sufficient homogeneity can be generated to justify mass analyses using whole cell lysates. Based on previous work with mouse embryonic stem cells (Bibel et al., 2004), we found that selecting for the most rapidly dividing ES cells by repeatedly plating them at clonal density effectively selects for cells with a true embryonic stem cell character by eliminating those that have begun to differentiate (Bibel et al., 2004; Yazdani et al., 2012). The logic of this protocol is that the exposure of cells with identical characteristics to the same inducers is more likely to generate differentiated cells with similar characteristics than is the case with the mixed populations of stem cells used in traditional differentiation protocol.

**Figure 1:**
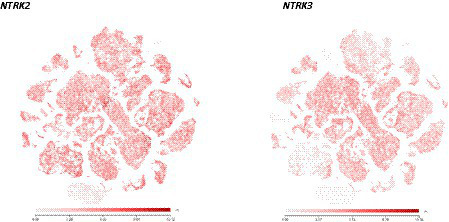
Scatter plots illustrating the similarities of *NTRK2* and *NTRK3* expression in the adult human brain. Most brain neurons express both receptors, raising the question as to how NT3 can selectively activate TrkC as it can also activate TrkB (see Ateaque et al., 2022 for a detailed account). *NTRK2*: https://celltypes.brain-map.org/rnaseq/human_m1_10x?selectedVisualization=Scatter+Plot&colorByFeature=Gene+Expression&colorByFeatureValue=NTRK2. *NTRK3*: https://celltypes.brain-map.org/rnaseq/human_m1_10x?selectedVisualization=Scatter+Plot&colorByFeature=Gene+Expression&colorByFeatureValue=NTRK3.

With this re-cloning strategy combined with differentiation inducers previously used (Arber et al., 2015; Telezhkin et al., 2016), essentially pure populations of GABA-ergic neurons could be generated from human ES cells (Merkouris et al., 2018). Importantly, essentially all cells are not only neurons, but also all express the BDNF/NT4 receptor TrkB and the NT3 receptor TrkC, and at a ratio similar to what is found in the human brain as determined by single cell RNAseq data (Ateaque et al., 2022 and see Figure 1). RNAseq data using RNA extracted from 1 month-old cultured neurons indicated negligible expression of the NGF receptor TrkA and of the neurotrophin receptor p75 (Merkouris et al., 2018; Ateaque et al., 2022), as is also the case in the human brain, with the notable exception of the basal forebrain cholinergic neurons. This pattern of neurotrophin receptor expression suggests that a major differentiation pathway may unfold under permissive culture conditions, in the absence of complex and changing cell–cell interactions taking place in the developing human brain. It is also of note that the differentiation procedure used did not involve BDNF or Trk activators that were omitted from the original protocol (Telezhkin et al., 2016). Indeed, neither TrkB nor TrkC showed any sign of activation (using antibodies reporting phosphorylated tyrosine residues of Trk receptors) in the absence of exogenously added neurotrophins (Ateaque et al., 2022). With regard to Trk receptor activation, it is interesting to note that according to a recent study comparing mouse embryonic neurons with neurons generated from human iPSCs, evolutionary conserved chromatin regions have been identified responding to BDNF by enhancer activation (Ibarra et al., 2022).

#### Activation of TrkB and TrkC in human neurons

**Figure 2:**
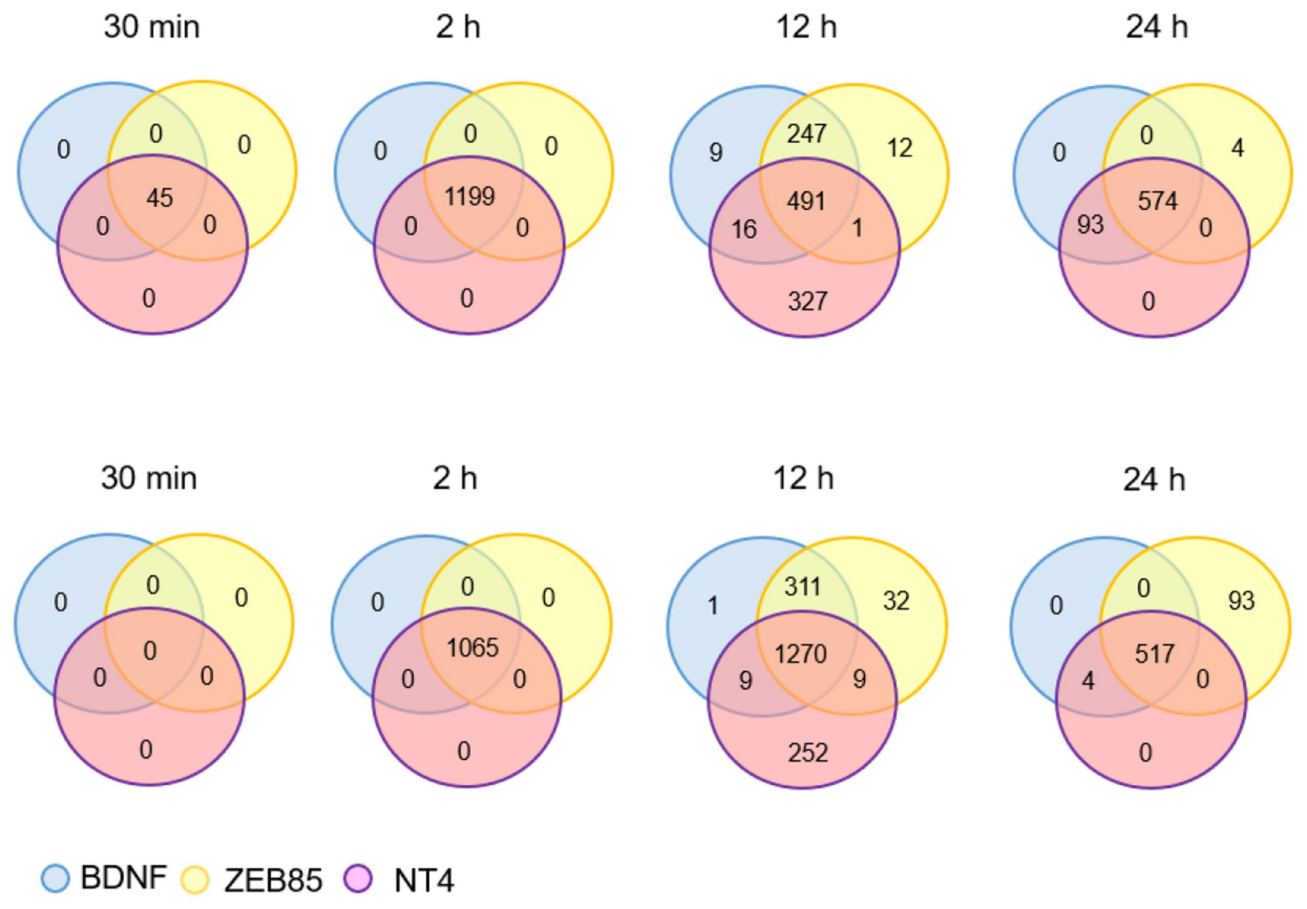
The numbers refer to the number of genes that were found to be up-regulated (upper row) or down-regulated compared to control (untreated cultures) at the indicated time points following addition of the 3 TrkB activators indicated. The genes were selected based on absolute fold-changes >2 and adjusted *p* value <0.01 cut-off criteria using the limma Bioconductor package in R software. This illustration is based on a recalculation of the results published in Merkouris et al. (2018) as explained in the text.

As could be expected from countless studies with rodent neurons, TrkB could be readily activated in cultured human neurons by both BDNF and NT4 (Merkouris et al., 2018). In addition, TrkB could be similarly activated by a TrkB agonistic antibody selected from a recombinant library designated ZEB85 (Merkouris et al., 2018). As the expression of endogenous neurotrophins in this *in vitro* system is negligible, no basal activation of Trk receptors was detected whereas the addition of any of these 3 TrkB ligands caused a rapid and robust TrkB phosphorylation signal as well as a large number of transcriptional changes detected by RNAseq using RNA extracted from lysed neurons as template (Merkouris et al., 2018). A re-analysis of these data using edge R and limma packages with the Bioconductor software that significantly reduces the rate of false positive genes (Zhang et al., 2014; Ritchie et al., 2015; Law et al., 2016) further revealed (Figure 2) that the transcriptional changes triggered by the 3 TrkB ligands are even more similar than those previously published (Merkouris et al., 2018). It is noteworthy that *NPAS4* is amongst the genes most strongly and rapidly up-regulated downstream of TrkB activation by BDNF, NT4 and ZEB85 or of TrkC by NT3 (Merkouris et al., 2018; Ateaque et al., 2022). *NPAS4* has long been recognized to be up-regulated by neuronal activity (Lin et al., 2008) and has been recently identified as key component of DNA repair (Pollina et al., 2023). Previous studies have indicated that increased transcription causes enhanced oxidative stress and DNA damage, suggesting a need for the activation of DNA repair mechanisms in postmitotic, long-lived cells such as neurons (Suberbielle et al., 2013; Madabhushi et al., 2015).

Similar to previous observations with rodent neurons (Frank et al., 1996; Sommerfeld et al., 2000), activation of TrkB with saturating ligand concentrations led to a prolonged loss of the phosphorylated-TrkB signal, for a period of at least 50 h. TrkB could not be re-activated by the re-addition of BDNF after this time, whereas activating TrkB with much lower initial, likely more physiological concentrations of BDNF such as 40 pM was sufficient as such to generate a clear Trk activation signal when adding the same low BDNF concentration just 24 h later (Ateaque et al., 2022). As TrkB is known to be also activatable by NT3, it was also important to determine whether NT3 could selectively activate TrkC in these neurons, given the co-expression of TrkB and TrkC by most CNS neurons. Elegant functional data gathered in the developing mouse cerebellum indicate that NT3 does exert a unique role with regard to dendritic arborization, not redundant with the role of BDNF also present in the very same structure (Joo et al., 2014). This apparent paradox can now be explained by the finding that TrkC can be selectively activated by very low, presumably physiological concentrations of NT3, without concomitantly activating TrkB. This conclusion was reached with help of ES cells engineered to suppress the activation of TrkB by extracellular ligands (Ateaque et al., 2022). This finding further supports the notion that *in vivo*, neurotrophins such as NT3 are only available in limiting quantities thus allowing selective receptor activation, in spite of TrkB and TrkC co-expression by most neurons. Interestingly, the downregulation of TrkB by BDNF does not seem to compromise the activation of TrkC by NT3 in the same neurons, indicating that the mechanisms involved are receptor-specific. Trk receptor downregulation following the use of saturating concentrations of TrkB ligand was also observed using saturating concentrations of NT3 (Ateaque et al., 2022). Perhaps most important in a therapeutic perspective, low, presumably physiological ligand concentrations allowed Trk receptor re-activation (Ateaque et al., 2022).

### Trkb activation by reagents others than neurotrophins

So far, the administration of BDNF to restore compromised neuronal function has not worked well in humans. Repeated failures triggered a number of attempts to develop small compounds able to activate TrkB, such as a BDNF loop 2 mimic designated LM22A-A, 7,8-dihydroxyflavone and the antidepressants Deprenyl and Amitriptyline. However, none of these agents turned out to activate TrkB in assays specifically designed to report TrkB activation (Boltaev et al., 2017). The reasons for these failures are unclear whereby in the meantime it has been reported that all known antidepressants bind to and activate TrkB in a BDNF-and cholesterol-dependent manner (Casarotto et al., 2021). This unexpected finding is all the more surprising given that none of the antidepressants reported to activate TrkB have been developed based on their TrkB-activating ability and that some are structurally unrelated. Meanwhile, we tested the ability of some of these compounds, including fluoxetine and imipramine for their ability to activate human TrkB in cultured neurons, both in the presence or absence of added BDNF, given that antidepressants are thought to potentiate the action of endogenous BDNF (Casarotto et al., 2021). The preliminary results turned out to be negative, also in the presence of exogenous cholesterol supplemented to the cultures (Ateaque et al., unpublished results). As the successful incorporation of cholesterol into neurons was not monitored, these negative results should not be overinterpreted. Also, the virtual absence of glial cells in our cultures and the immature connectivity between the neurons are likely to be complicating factors whereby the neurons were electrically excitable at the time when they were tested (Telezhkin et al., 2016). With regard to the activation of TrkB by macromolecules other than neurotrophins, a number of agonist antibodies have been developed causing TrkB dimerization and activation, in addition to ZEB85 discussed in the above (Bai et al., 2010; Traub et al., 2017; Guo et al., 2019). Importantly, some of these reagents have been already used in relevant animal models including glaucoma. These were shown to demonstrate positive effects with regard to the prevention of the death of retinal ganglion cells (Bai et al., 2010). The selectivity of such reagents for TrkB and their lack of activation of the neurotrophin receptor p75 pathway suggests that they may be promising reagents towards ameliorating a number of conditions in humans.

## Conclusion

Work with neurotrophins using animal models has revealed that this signalling system plays multiple roles in the nervous system. These models also provided important clues as to what to look for in humans. Further illustrating the consequences of the extraordinary early discovery of NGF, the first mutation in a human neurotrophin signaling gene linked the NGF receptor TrkA with pain insensitivity (Indo et al., 1996). This finding and a large body of related work with rodents led to the development of new approaches to treat chronic pain in humans using NGF neutralizing antibodies (Wise et al., 2021; Ma et al., 2022). While the work summarized in the above suggests that enhancing the BDNF/TrkB pathway would be desirable in a wide range of conditions, success has been quite limited thus far, in part because of the unfavorable pharmacodynamic characteristics of BDNF, its interaction with the neurotrophin receptor p75 and the long-lasting downregulation of its receptor TrkB caused by saturating ligand concentrations used in most *in vivo* studies (Miranda-Lourenco et al., 2020). Test systems based on the generation of human neurons from stem cells should now facilitate the selection of suitable drug candidates and lead to a better understanding of Trk receptor down-regulation and of its prevention. Last, the delineation of suitable clinical indications will be helped by the availability of sensitive and specific methods to measure BDNF levels in body fluids more directly relevant to the function of the nervous system than has been the case so far.

## Author contributions

All authors listed have made a substantial, direct, and intellectual contribution to the work and approved it for publication.

## Footnotes

^1^ https://www.proteinatlas.org/ENSG00000176697-BDNF/brain

^2^With regard to the expression of BDNF, TrkB and the neurotrophin receptor p75 across a number of developing mammals, the reader is referred to a recent review by Esvald and colleagues (Esvald et al., Frontiers in Molecular Neuroscience *in press*).
